# Surgery of the Liver and Gall Bladder

**Published:** 1906-01-13

**Authors:** 


### SURGERY OF THE LIVER AND GALL BLADDER.

**Cholelithiasis.**—Our knowledge of gall-stone disease—and especially the part which infection takes therein—has greatly increased during the past few years. As Wiener[1] says, we are no longer content to make a diagnosis simply of gall-stones. We must determine the location of the stone or stones in the biliary tract, the degree of inflammation present, and whether there is partial or complete obstruction to the flow of bile before we can decide whether medical or surgical treatment is to be instituted. The ideal treatment of this class of cases is to treat them medically during a few attacks. If one or more small stones are passed, followed by relief of the pain, then an operation is not indicated, because nature, by expelling all the stones present, may bring about a cure. If, on the other hand, one or more severe attacks of colic are not accompanied by the passage of any stones per anus, then an operation is indicated, and indicated long before there has ever been any jaundice.

In 80 per cent. of gall-stone attacks there is no jaundice. The finding of stones in the stools is rare, because only in exceptional cases do the stones leave the gall-bladder during an attack. There may be a large stone in the common duct, and yet there may be no icterus at all. In cases of chronic obstruction of the common duct it is important to ascertain if the obstruction is due to tumour or to stone. If due to stone, the gall-bladder is generally shrunken, not palpable, the icterus moderate and varying, the stools at times brown, at other times grey, the colic of varying degrees of severity, the spleen generally enlarged, and the fever intermittent. If, on the other hand, the obstruction is due to cancer, the gall-bladder is enlarged, often palpable, the icterus is continuous and progressive, the stools are constantly grey, and there is generally an absence of colics, of enlargement of the spleen, and of fever. Common as gall-stones are, in 95 per cent. of the cases they cause very mild symptoms or none at all. Kehr gives the following positive indications for operation: (1) Acute sero-purulent cystitis and pericystitis; (2) adhesions resulting from a pericystitis, if symptoms are present (pain, pyloric or duodenal stenosis, ileus, etc.); (3) chronic obstruction of the common duct; (4) chronic obstruction of the cystic duct; (5) all cases that begin mildly, but are progressive, do not improve under treatment, and which incapacitate the patient from enjoying life or attending to his affairs; (6) purulent cholangitis and liver abscess; (7) perforation of gall-bladder and peritonitis; (8) gall-stone morphinism. In rare instances, where there have been colics present before operation, no stones are found. The gall-bladder is found distended with turbid bile, and its mucous membrane is inflamed. There are cases of cholecystitis not due to cholelithiasis, but to an infection pure and simple, probably from the gut. The whole trend of modern progressive surgery is towards the removal not only of the gall-stones, but also of the diseased organ in which they develop—the gall-bladder. Primary cholecystectomy is to-day the operation of choice in the large majority of cases of gall-stones because: (1) The mortality is very small, lower even than after cholecystostomy; (2) the wound heals much more rapidly; (3) there is no danger of recurrence, either of the cholecystitis or of the formation of new stones; (4) no secondary operations are necessary. Naturally, stones in the common or hepatic ducts should be removed at the same time with the gall-bladder, and, if necessary, drainage instituted either through the stump of the cystic duct or through the common or hepatic duct.

[1] Med. Rec., July 8, 1905, p. 54.

